# Randomized controlled trial comparing 400μg sublingual misoprostol versus placebo for prevention of primary postpartum hemorrhage

**DOI:** 10.11604/pamj.2020.36.186.22538

**Published:** 2020-07-15

**Authors:** Rym Zgaya, Imen Ghadhab, Mohamed Amine Triki, Raja Briki

**Affiliations:** 1 Department of Gynecology and Obstetrics, Farhat Hached University Hospital, Sousse, Tunisia,; 2 Faculty of Medicine, Sousse, Tunisia

**Keywords:** Postpartum hemorrhage, maternal mortality, misoprostol, prevention, uterotonics, side effects

## Abstract

**Introduction:**

obstetric hemorrhage is estimated to cause 25% of all maternal deaths and is the leading direct cause of maternal mortality worldwide. The World Health Organization recommended the use of uterotonics that should be offered for all women who will give birth but in some countries or in special situations oxytocin is not available. The goal of this study is to determine whether the 400μg dose of Misoprostol decreases the incidence of postpartum hemorrhage (PPH) of women who did not show signs of hemorrhage.

**Methods:**

a prospective randomized double blind controlled trial was conducted between February 2012 and June 2012, among women in the active stage of labor attending the Obstetric Gynecology Department, University Hospital Farhat Hached of Sousse, Tunisia. Women with term singleton pregnancies greater than 32 weeks of amenorrhea with anticipated vaginal delivery were eligible for the study. Participants were randomly assigned to receive 400 μg sublingual Misoprostol or 2 ets of placebo immediately after cord clamping. The primary outcome measures were an estimation of blood loss including the subjective finding of vaginal hemorrhage > 500 ml, the decrease of hemoglobin and hematocrit, a change in hemodynamic parameters, and the need for additional dose of oxytocin. Secondary outcomes were occurrence of possible side effects such as: headache, nausea, vomiting, pyrexia, diarrhea and abdominal pain.

**Results:**

a total of 211 patients were randomized: 111 in the Misoprostol group (Cytotec*) and 100 patients in the placebo group. The two groups were similar in terms of sociodemographic characteristics. Significant difference between the 400-μg of Misoprostol and placebo group were recorded in mean postpartum blood and PPH occurrence. The difference in pre- and postpartum hemoglobin loss (expressed in grams per 100 ml) was 1.21 ± 1.05 for the Misoprostol group and 1.51 ± 0.74 for the placebo group with significant difference (p = 0.02). No differences were observed in the occurrence of headache, dizziness, vomiting, diarrhea and metallic taste but the incidence of shivering was more than twice as great among women receiving Misoprostol than among those treated with placebo with a significant difference (p = 0.01). Similarly, women who received Misoprostol had a significantly higher mean temperature after delivery in comparison with those receiving placebo.

**Conclusion:**

misoprostol, administered as 400 μg after delivery, appears to be effective for the prevention of post-partum hemorrhage, but its side effects appears to be significant.

## Introduction

Maternal mortality rate is still high worldwide. According to World Health Organization (WHO), severe bleeding is responsible for 25% of the 500,000 annual maternal deaths and is involved in 4-5% of deliveries [1], and postpartum hemorrhage (PPH) is the leading cause of maternal mortality that results from bleeding. The large majority of them in developing countries. In our country and according to a report of the National Committee on Mortality in 2010 [2], maternal mortality represents 44.8/100,000; eighty percent live births in hospitals, half of which (49%) is due to PPH. The most effective method for reducing PPH is the active management of the third phase of delivery requiring prophylactic utero-tonic drugs, the management of which is still ineffective in less favored areas where prevention is most needed. Injece oxytocin and/or Ergometrine are the most widely used agents Methylergometrine (Methergin) is less used because of its sometimes serious cardiovascular adverse effects [3]. They require parenteral administration, and therefore skills to give injections as well as sterile needles and syringes. In addition, Ergometrine requires refrigeration and oxytocin may be inactivated if exposed to high ambient temperatures. For this reason, the use of misoprostol to prevent PPH has attracted considerable attention [4]. Several authors have evaluated misoprostol for the prevention of postpartum hemorrhage, attracted by its low price, stability at room temperature and easy way of use and compared rectal misoprostol to Syntometrine oxytocin 2 IU and ergometrine 0.5mg. and concluded that postpartum blood loss and changes in hemoglobin levels were similar in the both groups [5]. The same authors in another publication [6,7], compared rectal and oral misoprostol with placebo. No significant differences were found with either misoprostol regimen when compared with placebo these studies contended that misoprostol was promising in the prevention of PPH and had the advantage of lower cost in comparison with syntometrine. FIGO (International Federation of Gynecology and Obstetrics) admits in its latest guidelines, published in May 2012, a 600 μg dose of sublingual Misoprostol significantly reduces the risk of delivery hemorrhage in the absence of oxytocin, which is the case in the majority of underdeveloped countries. This dose of Misoprostol (600μg) is not devoid of any adverse effects according to FIGO: shivering in 18% to 52% of cases, fever in 5% of cases and gastrointestinal disorders (diarrhea, nausea, vomiting) in 1% of cases [7]. We proposed to conduct a randomized clinical trial to determine if a dose of 400μg of sublingual Misoprostol will result in a significant decrease in the incidence of delivery hemorrhage and reduce blood loss, on the one hand, (which constitutes an economic gain) and if this dose will reduce the effects unwanted on the other hand; and this in a population at low risk of bleeding.

## Methods

A prospective randomized double blind controlled trial was conducted between February 2012 and June 2012, among women in the active stage of labor attending the Obstetric Gynecology Department, University College Hospital Farhat Hached of Sousse, Tunisia. Women with term singleton pregnancies greater than 32 weeks of amenorrhea with anticipated vaginal delivery were eligible for the study. The exclusion criteria were patients at high risk for postpartum hemorrhage: coagulation disorders, a placenta Previa, a placental retro hematoma, a HELLP syndrome, in utero fetal death, maternal fever (≥38°C), prolonged Labor (> 12 hours) and need for cesarean delivery. Patients with hypertensive diseases in pregnancy, anemia (hb < 8), prepartum hemorrhage, previous history of uterine rupture, or conditions requiring prophylactic oxytocin infusion after delivery (e.g. multiple pregnancy, previous history of PPH) were also excluded. All patients were adequately counseled and voluntary informed consent was obtained before recruitment to the study. After providing informed consent and reaching the second stage of labor, the randomization was done by computer and the result is marked on a card kept by a third person. Each patient was included in one of two groups: 200μg of misoprostol (A) or Placebo (B). Placebo ets were with a similar appearance to that of the misoprostol ets used, which meant that patients in both groups received two ets. Patients are recruited from the reception area and checked to see if they have one or more exclusion criteria that exclude them from the study; we make them sign an informed consent. A blood sample is taken if the patient has no initial NFS test. Surveillance is carried out by all midwives and interns.

## Results

**Figure 1:**
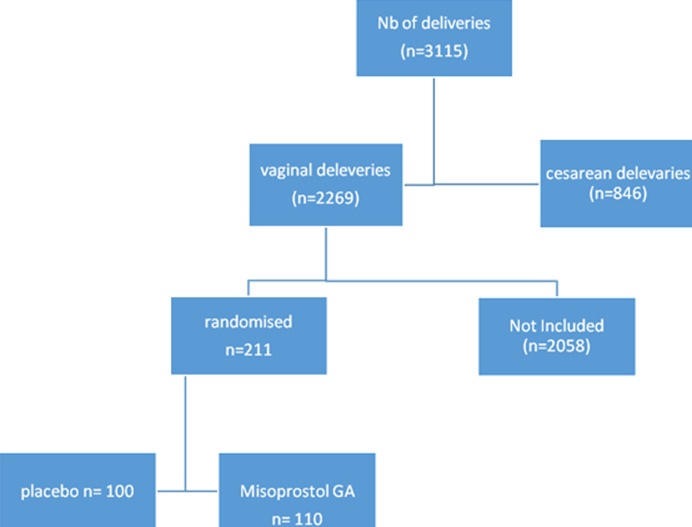
schema of the clinical study specifying the distribution of the patients in the 2 groups

Three thousand one hundred and fifteen cases of deliveries occurred during the study period; of which 2269 gave birth vaginally (72.8%) 2058 deliveries were not randomized either because they were not available or because they did not meet the inclusion criteria. A total of 211 patients were randomized: 111 in the Misoprostol group (Cytotec*) and 100 patients in the placebo group (Figure 1).The two groups were similar in terms of sociodemographic characteristics, age, educational level, socioeconomic condition, gynecological history of parturient (Table 1). Additionally, the term of pregnancy, the nature of the work (spontaneous or triggered), the dose of Prepidil gel administered, the amount of syntocinon administered, the premature rupture of the membranes and the mode of delivery did not differ significantly between the groups. The postpartum hemorrhage rate is lower in the Misoprostol (G1) group where 6 cases were reported, a rate of 5.5% versus 15 women, or a rate of 15% in the placebo group with a statistically significant difference (p = 0.021). The pattern was placental retention in 10 cases 2 in the Misoprostol group (G1) versus 8 in the placebo group (G2), with 6 cases of uterine atony in the placebo group (6%) and 2 in the Misoprostol group (1.8%) were reported. These hemorrhages were curbed by medical treatment. Artificial delivery with uterine revision was performed in case of placental retention. None of these patients required a blood transfusion. It should be noted that hematological characteristics prior to delivery are statistically similar for hematocrit and hemoglobin. The percentage hematocrit drop was respectively in the Misoprostol (G1) group and the placebo (G2) group of 3.33% ± 0.33 and 4.29% ± 0.29 with a significant difference (p = 0.033). The difference in pre- and postpartum hemoglobin loss (expressed in grams per 100 ml) was 1.21 ± 1.05 for the Misoprostol group and 1.51 ± 0.74 for the placebo group with significant difference (p = 0.02). No significant difference found between the two study groups with regard to blood pressure or changes in heart rate. No differences were observed in the occurrence of headache, dizziness, vomiting, diarrhea and metallic taste but the incidence of shivering was more than twice as great among women receiving misoprostol than among those treated with placebo with a significant difference (p = 0.01). Similarly, women who received misoprostol had a significantly higher mean temperature after delivery in comparison with those receiving placebo. There were 14 cases of abdominopelvic pain in the group Misoprostol or 13% and 6 cases the placebo group is 6%, the difference is significant (p = 0.035) and 29 cases of nausea in the Misoprostol group, 26.4% versus 8 cases in the placebo group or 8% .

**Table 1:** maternal characteristics in both study groups

Characteristics		M		P		P	
		G1		G2			
		average	standard deviation or%	average	standard deviation or%		
**Age**		29,73	± 6,3	30,13	± 5,99	0,89	NS
**School level**	**illiterate**	5	4,60%	3	3,10%	0,45	NS
	**primary level**	21	4,60%	25	25,50%		
	**Secondary level**	71	19,30%	64	65,30%		
	**Higher level**	12	65,10%	6	6,10%		
**Socio-economic condition**	**bad**	8	11,00%	6	6,10%	0,791	NS
	**medium**	99	90,80%	91	92,90%		
	**correct**	1	0,90%	1	1,00%		
**Gravidity**		2,1	± 1,55	2,23	± 1,58		
**Parity**	**0**	5,3	48,60%	40	41,20%	0,49	NS
	**1**	29	26,60%	28	28,90%		
	**2**	17	15,60%	14	14 ,40%		
	**≥3**	10	9,20%	15	15,50%		
**Scarred uterus**		3 femmes		1 femme		0,64	NS

**NS:** No Significant; **G1:** M: Misoprostol; **G2**: Placebo

## Discussion

Obstetric hemorrhage is estimated to cause 25% of all maternal deaths and is the leading direct cause of maternal mortality worldwide [8]. In Africa and Asia, nearly one-third of pregnancy-related deaths are associated with postpartum hemorrhage [9]. The World Health Organization (WHO) and other international agencies recommend that all women giving birth receive uterotonics during the third phase of labor for the prevention of postpartum hemorrhage (active management of the third phase of childbirth). It should be offered for all women who will give birth. It involves interventions to facilitate delivery of the placenta by increasing uterine contractions and preventing PPH by avoiding uterine atony. The first goal of this study is to determine whether the 400ug dose of misoprostol decreases the incidence of PPH by determining the proportion of women who did not show signs of hemorrhage. Classically, the diagnosis of PPH is made subjectively when the surveillance of the woman giving birth in the instants which follow the birth, notes blood losses more abundant than the “normal”. Another way of assessing postpartum blood loss is the comparison of hemoglobin and hematocrit at admission to the work room with those measured on the 3rd day postpartum. Other parameters that can assess the importance of bleeding are: the change in hemodynamic constants (blood pressure and pulse before and after delivery), the need for other interventions (Use of additional uterotonics, blood transfusions, manual delivery of placenta, surgical procedures for HPP treatment (eg hysterectomy), and status of the uterine globe (hardness). In our study the proportion of women who did not have hemorrhage was 94.5% in the misoprostol group, 85% in the control group. The difference between the 2 groups is significant. The drop in hemoglobin in g/100ml tends to be greater in the placebo group (1.51 ± 0.74 versus 1.21 ± 1.05 with a significant difference p = 0.02). The hematocrit falls were respectively (in %) in the misoprostol group and the placebo group of 3.33 ± 0.33 and 4.29 ± 0.29 was significant (p = 0.033). For blood pressure there was no difference between the 2 groups in pre- and post-delivery. The proportion of women with flaccid uterus was 40% for the placebo group and 40% in the misoprostol group. The proportion of women with placental retention was 53% for the 40% control group in the misoprostol group. The percentage of women who received at least one treatment for hemorrhage was 5.4% in the misoprostol group and 15% for those who received nothing. This difference is significant (p = 0.021) while the proportion of women who received an uterotonic to treat hemorrhage is 4.5% in the misoprostol group and 13% in the placebo group. This difference is not significant (p = 0.054) and none of the 2 groups had a blood transfusion to treat bleeding.

The proportion of women who received manual delivery of the placenta to treat hemorrhage was 5.4% in the misoprostol group and 11% for those who received nothing. The provider performs a manual delivery of the placenta in the event of a finding of placental retention. Only one woman, in each of the 2 groups, underwent surgery to treat the bleeding. The file of the 2 patients shows that a cervical suture was recommended to stop the bleeding. A study conducted in the province of Pakistan [10] in remote mountain villages, where the majority of births are performed at home by trained traditional birth attendants and where access to maternal health emergency services is limited. A randomized, double-blind study was conducted in approximately 1400 women and was designed to test whether 600μg of oral misoprostol reduced the incidence of PPH when administered during the third phase of labor. The authors concluded that: compared with placebo, oral misoprostol reduced the incidence of HPP by 24%. Similarly, women who received misoprostol experienced a lower decline in hemoglobin (>2g/dL) after delivery. Among others, in the Cochrane library [11] a study including 72 trials (52,678 women). Oral or sublingual misoprostol compared to placebo is effective in reducing severe PPH and need for blood transfusions. Compared with conventional injece uterotonics, oral misoprostol was associated with increased risk of severe PPH and administration of additional uterotonics, but with a declining trend in blood transfusion requirements. Joy SD *et al*. analyzed all published studies [12] (from January 1996 to May 2002) evaluated the effectiveness of misoprostol in minimizing blood loss during the third phase of labor. Seventeen studies included 28,170 subjects, of whom about half received misoprostol and the remainder received either placebo or other uterotonic. When evaluating studies comparing misoprostol to placebo, those who received oral misoprostol had a decreased risk of additional need for uterotonics (OR 0.64, 95% confidence interval 0.46, 0, 90). In contrast, in studies comparing misoprostol with oxytocin, oxytocin was associated with significantly lower rates of postpartum hemorrhage. In studies comparing misoprostol to Syntometrine, misoprostol has been associated with higher rates of additional uterotonic need. Since adverse effects vary with the dose administered, research should focus on the evaluation of the lowest effective dose for routine use as well as for optimal administration. Neither intramuscular prostaglandins nor misoprostol are preferable to conventional injece uterotonics as part of delivery management.

In the satisfactory meta-analysis of G Justus *et al*. [13], conducted to collect randomized trials contributing to indirect and direct comparisons; there is no evidence that the dose of 600μg is more effective than 400 μg to prevent blood loss = 1000ml. When comparing misoprostol versus other uterotonics, it was rather disappointing to verify that the use of Misoprostol, at a dose of 600 to 800μg, is less effective than other. In addition, the results obtained with 400-500 mg of misoprostol did not differ significantly from those obtained with 600μg. Interestingly, in two recent studies in misoprostol versus conventional uterotonics [14], Misoprostol, when combined with oxytocics, further accentuated the reduction in the prevalence of severe hemorrhage, as well as hemorrhage from delivery in general. These associations, however, led to an even greater incidence of side effects such as tremors and fever. But would it not be more “accepe” in high risk hemorrhagic situations, if the efficiency gain was significant. The joint administration of prostaglandins E1 and oxytocin could therefore usefully be the subject of further studies to evaluate this surplus efficiency. On our side we showed that compared to placebo there is a reduction of post partum hemorrhages through a subjective assessment of bleeding and the difference of Hb and Ht in pre and post partum. By continuing to weigh the benefits of one drug against another, the distribution of misoprostol must continue in developing countries, where it is the only choice and should be measured against no treatment at all. Regarding adverse effects, in our study the percentage of women who had shivering group M 35.5% while it is 12% in the placebo group with a significant difference (p = 0.01). The percentage of fever is greater in the group M 9.1% vs 1% with a significant difference (p = 0.01). We found abdominopelvic pain in 13% of cases in the misoprostol group against 6% in the P group, the difference is significant (p = 0.035). For nausea, 26% of cases were found in group M vs. 8% in the other group with a significant difference (p = 0.01). There was no significant difference between the 2 groups regarding headache, vertigo, vomiting, diarrhea and metallic taste. In the 2012 Cochrane Library [11] including 72 trials, a significant increase in shivering and a temperature of 38°C was found in the misoprostol group compared with placebo and others. The method used to measure blood loss can be criticized because it was limited to the length of time women stayed in the delivery room, which explains its lower average value compared to that reported by other authors. The visual subjective method used to assess blood loss generally underestimates losses [15,16]. Descargues G *et al*. in a retrospective study of 5,517 natural deliveries showed that the quantification of bleeding visualization losses was a very poor indicator, especially when there was no way to collect bleeding.

## Conclusion

Thus, many studies have found that Misoprostol is a little less effective than oxytocin. This has had the effect of reducing the image of this product, despite its glory especially in disadvantaged areas. The main conclusions are to use oral misoprostol at a dose of 400 microgram for the prevention of post-hemorrhage Partum in centers where oxytocin is not available. On the other hand side effects of misoprostol persist despite a reduction in dosage. However, these side effects are not severe. We think it was more interesting to expand the sample and involve other health facilities and other health providers.

### What is known about this topic

- The World Health Organization recommended the use of uterotonics for all women who will give birth.

### What this study adds

- Our study makes it possible to highlight the place of misoprostol in the prevention of the hemorrhage of the delivery especially in the cases when the uterotonics are not available.
